# Leydig Cell Tumor in a Patient with 46,XX Disorder of Sex Development (DSD), Ovotesticular: A Case Report and a Review of the Literature

**DOI:** 10.1155/2021/5552305

**Published:** 2021-03-29

**Authors:** Steffen Gretser, Maria-Noemi Welte, Frederik Roos, Jens Köllermann

**Affiliations:** ^1^Dr. Senckenberg Institute of Pathology, University Hospital, Goethe University Frankfurt am Main, Frankfurt am Main, Germany; ^2^Department of Urology, University Hospital, Goethe University Frankfurt am Main, Frankfurt am Main, Germany

## Abstract

Disorder of sex development (DSD) is a rare condition with atypical development of chromosomal, gonadal, or anatomical sex. It is classified in different subgroups based on the patient's karyotype, gonadal dysgenesis, and the appearance of the internal and external genitalia. Within the subgroups, the risk for developing neoplasms varies a lot. Here, we report the case of a 41-year-old patient with disorder of sex development, showing a 46,XX karyotype with an ovotestis and the simultaneous manifestation of a Leydig cell tumor in the ovotestis. The patient initially presented with infertility, and a suspicious lesion of the left testicle was noted on MRI-Scan. Upon resection, a Leydig cell tumor and an ovotestis were diagnosed. Nongerm call tumors are rare in patients with DSD. We report a nongerm cell tumor in a patient with 46,XX DSD, ovotesticular. This shows that although 46,XX DSD, ovotesticular is known to have a low potential for germ cell neoplasia, nongerm cell tumors can develop and should be into account for the management of those patients.

## 1. Introduction

The international consensus statement defined disorders of sex development (DSD) as “congenital conditions in which development of chromosomal, gonadal or anatomical sex is atypical” [1]. In that consensus statement, a simple classification of DSD in three core categories was introduced: sex chromosome DSD, 46,XY DSD, and 46,XX DSD. Besides the karyotype, gonadal dysgenesis and the appearance of the internal and external genitalia are used to further stratify these categories. The term gonadal dysgenesis is used to describe the incomplete or defective function of the gonads, including both ovary and testis. It is characterized by varying levels of immaturity and dysfunction and can be divided into complete or partial dysgenesis [2]. In complete gonadal dysgenesis, the gonads can either be missing completely or show a complete insufficiency. In partial gonadal dysgenesis, there is an incomplete gonadal differentiation resulting in mixed gonads (e.g., unilateral testis and contralateral streak gonad) or ovotestis. In ovotestis, testicular and ovarian tissues are present in the same gonad. This condition can be bilateral, but the contralateral side can also show either testicular or ovarian tissue [3].

Sex chromosome DSD includes numerical alterations in the sex chromosome (e.g., Turner syndrome (45,X), Klinefelter's syndrome (46,XXY)) and mosaic karyotypes with mixed gonadal dysgenesis. 46,XY DSD includes subtypes with complete gonadal dysgenesis, partial gonadal dysgenesis (ovotestis), and a number of endocrine/hormonal defects (e.g., complete androgen insensitivity) [1].

46,XX DSD can be separated in three subgroups. The largest group consists of 46,XX DSD patients with androgen excess caused by various conditions, for example, congenital adrenal hyperplasia. This accounts for >90% of the cases with 46,XX DSD.

The second group contains unclassified disorders, for example, the Mayer-Rokitansky-Küster-Hauser syndrome [4]. The last group contains disorders of gonadal development. It contains 46,XX DSD with gonadal dysgenesis, 46,XX DSD testicular, and 46,XX DSD ovotesticular. In 46,XX DSD with complete gonadal dysgenesis, patients are found to have a primary ovarian insufficiency with reduced germ cells in the ovary. In 46,XX testicular DSD, patients show normal male internal genitalia and normal/ambiguous external genitalia. 46,XX ovotesticular DSD describes patients with a 46,XX karyotype and the presence of ovotesticular gonads. This category accounts for 60% of all ovotesticular disorders. In this subgroup, translocation of the sex-determining region of the Y-chromosome (SRY) and mutations in several genes have been described in literature [5]. These include protesticular genes like *FGF9*, *DMRT1*, *SOX9*, and other members of the *SOX* family, proovarian genes like *RSPO1*, *FOXL2*, and *WNT4*, and genes with mixed or unknown pathogenic mechanism such as *NR5A1*, *WT1*, and *NR2F2* [6].

## 2. Case Presentation

**Figure 1:**
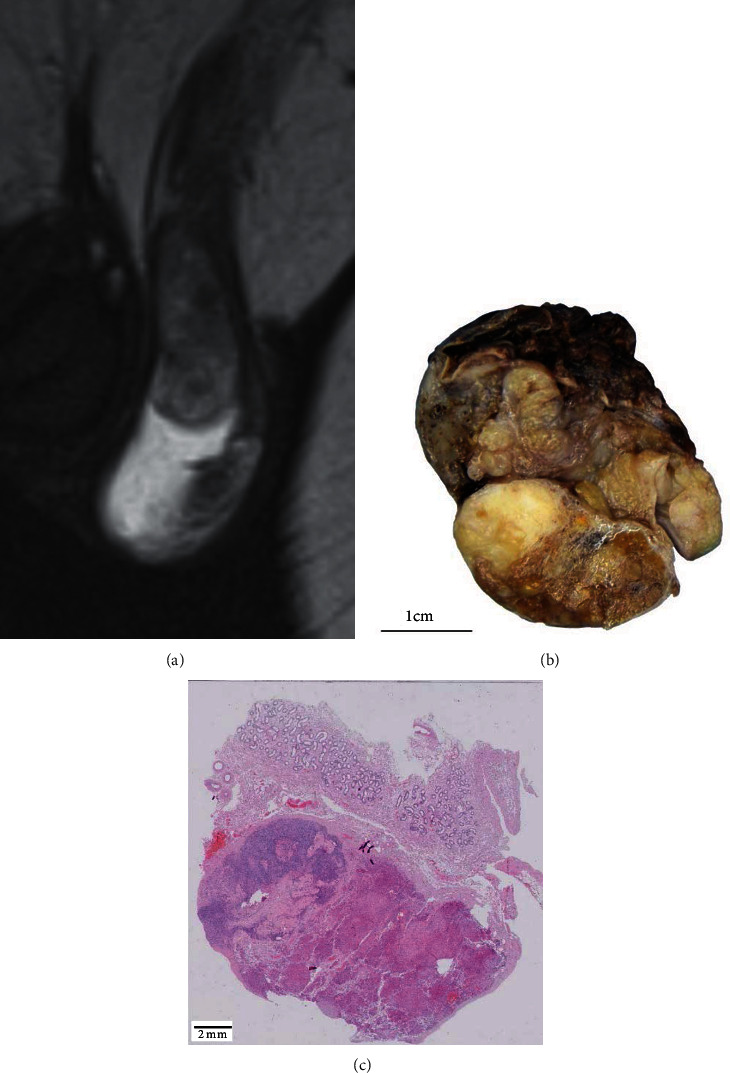
MRI-Scan showing a suspicious lesion of the remaining left testis (a). Cross-section of the specimen showing a biphasic lesion (b). A microscopic overview showing paratesticular tissue at the top, ovarian tissue on the left side, and an eosinophilic tumor on the right side (c).

We report the case of a 41-year-old patient who was admitted to our urological department for further investigation of a suspicious lesion of the left testicle noted on MRI-Scan (Figure 1(a)) and infertility.

The history is significant for inguinal cryptorchidism on the right side, treated by orchiectomy. The patient is known to have a 46,XX genotype, but his sexual identification is male. Both the karyogram and the orchiectomy were performed abroad, and no information including the histology of the right testis or the presence of the SRY is available.

**Figure 2:**
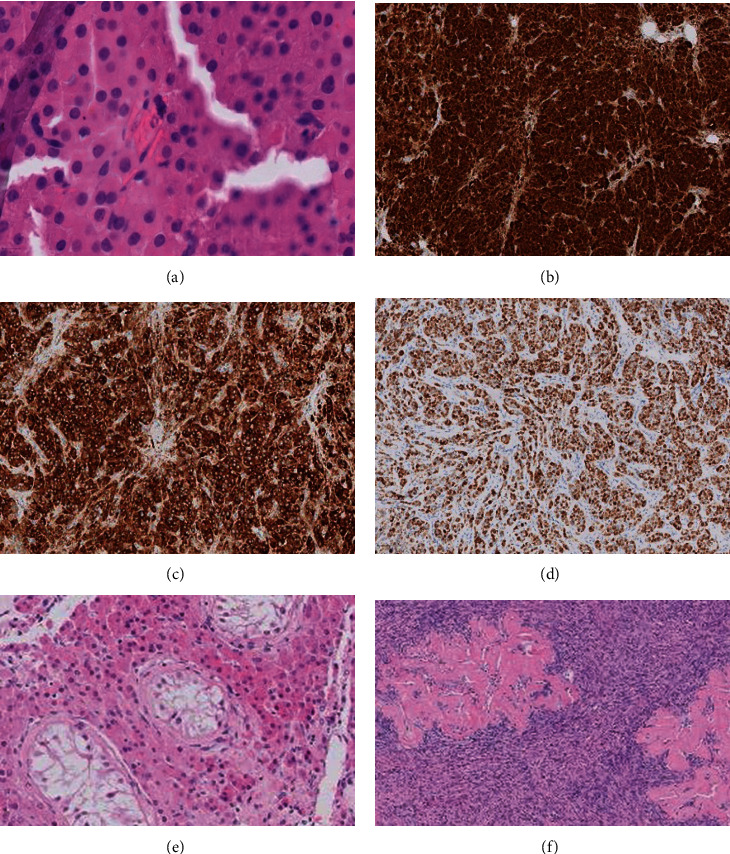
Leydig cell tumor with characteristic Reinke crystalloids (a), positivity for calretinin (b), inhibin (c), and melanA (d). Compressed seminiferous tubules in the Leydig cell tumor (e). Ovarian tissue with corpora albicantia (f).

On clinical examination, the patient showed a scrotal hypospadias with an elevated left testis which was inconspicuous on palpation. Further investigation showed azoospermia with hypergonadotropic hypogonadism (LH: 31 IU/L (ref. 1,7-8,6); FSH 44 IU/L (ref. 1,5-12,4); testosterone 226 ng/dL (ref. 249-836)). Tumor markers were negative (hcg+*β*hcg 1,0 IU/L (ref. <5); AFP 7,1 (ref. <7)).An orchiectomy was performed, and the specimen was submitted for pathological investigation. Macroscopically, a biphasic mass was observed (Figure 1(b)). Histologically, the main tumor mass showed a solid growth pattern (Figure 1(c)) with eosinophilic granules and occasional Reinke crystalloids (Figure 2(a)). Immunohistochemic positivity for inhibin, calretinin, and melanA confirmed the diagnosis of a Leydig cell tumor (Figures 2(b)–2(d)). A few remaining compressed seminiferous tubules without remaining spermatogonia were embedded in the tumor (Figure 2(e)). The absence of mitosis, a low Ki-67 index (1-2%), and p53 wild type favoured a benign behavior. A germ cell neoplasia in situ was excluded on the basis of HE-morphology and additional Oct3/4 stains. The second mass showed ovarian stroma with several corpora albicantia, but without any oocytes or follicles, resulting in the initial diagnosis of a DSD, ovotesticular (Figure 2(f)).

## 3. Discussion

Patients with DSD are known to have an increased risk for gonadal neoplasia, namely, germ cell neoplasia (germ cell neoplasia in situ, gonadoblastoma, and seminoma/dysgerminoma). The type of DSD is an important variable in the development of gonadal neoplasia [7]. As specified in the consensus statement, the risk for germ cell tumors is highest in patients with gonadal dysgenesis in general with the exception being ovotesticular DSD [1]. The mechanisms behind the increased risk for germ cell neoplasia in DSD are complex and not yet fully understood. An inappropriate or defective microenvironment with disturbed maturation of germ cells appears to play a major role in the development of germ cell neoplasia and its precursor lesions in DSD [8]. In this condition, abnormal expression of Oct3/4 (POU5F1) and TSPY is seen in patients with DSD [9]. Oct3/4 (POU5F1) is linked to prolonged survival of the germ cells and established as a factor in the development of germ cell neoplasia. TSPY appears to be involved in mitotic proliferation but is also expressed in regular spermatogonia [8]. Treatment of patients with DSD is complex, and issues such as gender identity of the patient as well as the risk for germ cell neoplasia should be discussed in an interdisciplinary setting [1].

Nongerm cell tumors in gonads of patients with DSD are very rare. There are single reports of Leydig cell tumors in 46,XX patients with androgen excess caused by adrenal hyperplasia [10] and 46,XX DSD, testicular [11].

We report—to the best of our knowledge—the first nongerm cell tumor in a patient with 46,XX DSD, ovotesticular. This shows that although 46,XX DSD, ovotesticular is known to have a low potential for germ cell neoplasia, nongerm cell tumors can develop. Although Leydig cell tumors are most likely benign, they should be taken into consideration in the management and screening of patients with 46,XX DSD, ovotesticular.

## Data Availability

All supporting data (macroscopic and microscopic imaging) are included in the manuscript.
